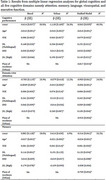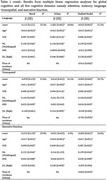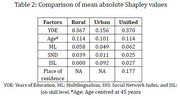# A unified measure for cognitive reserve that transcends rural and urban populations: Insights from two aging cohorts in southern India

**DOI:** 10.1002/alz70860_106281

**Published:** 2025-12-23

**Authors:** Ramya Burra, Jonas S Sundarakumar, Palash K Malo, Hitesh Pradhan, Raghav Prasad, Pooja Rai, Thomas Gregor Issac, Siva Athreya, Rajesh Sundaresan, CBR‐SANSCOG & CBR‐TLSA Study Teams

**Affiliations:** ^1^ Centre for Brain Research, Indian Institute of Science, Bangalore, Karnataka, India; ^2^ International Centre for Theoretical Sciences, Bangalore, Karnataka, India; ^3^ Indian Institute of Science, Bangalore, Karnataka, India

## Abstract

**Background:**

Cognitive Reserve (CR) refers to the ability to maintain cognitive and functional abilities despite brain aging, injury, or pathology. Attempts to estimate CR have mostly come from research in developed countries and from homogenous populations, which may not apply to other social and cultural contexts. Our study aims to fill this gap by measuring CR across two diverse aging populations, rural and urban, in southern India.

**Methods:**

We utilized cross‐sectional baseline data from two ongoing longitudinal aging cohorts, Centre for Brain Research‐Srinivaspura Aging, NeuroSenescence and COGnition (CBR‐SANSCOG, *n* = 4459) and CBR‐Tata Longitudinal Study of Aging (CBR‐TLSA, *n* = 663), in rural and urban India, respectively, comprising dementia‐free participants aged 45+ years. We used years of education (YOE, in years), job skill level (JSL, high or low), social network diversity (SND, number of active networks) and multilingualism (ML, single or multiple) as factors to estimate CR. We assessed cognitive performance using a culturally adapted computerized neuropsychological battery (COGNITO) in the domains of attention, memory, language, visuospatial, and executive function. We calculated overall cognitive performance by averaging standardized domain‐wise scores. We fitted, across rural and urban, separate and unified linear regression models. For the unified model we used the standardized scores of cognitive performances relative to each cohort as the target and addressed class imbalance using weighted regression with sample‐size normalization. We used SHapley Additive exPlanations (SHAP) to estimate the contribution of each factor.

**Results:**

The separate models led to similar factor contributions in the two cohorts. We therefore propose a unified measure for CR after adjusting for age and place of residence (rural/urban). The unified CR measure is: 0.079×YOE + 0.143×JSL + 0.025×SND + 0.192×ML. YOE and ML contribute the most with mean absolute SHAP: 0.370 for YOE, 0.062 for ML, 0.033 for SND, and 0.027 for JSL.

**Conclusions:**

This is the first study that estimated CR in the Indian context across two diverse, community‐dwelling, aging populations – rural and urban – accounting for multilingualism. As YOE is the most important factor contributing to CR in both cohorts, enhancing levels of early educational attainment through public policies could help lower dementia risk in India.